# Effectiveness and acceptability of ventilation modifications in healthcare facilities, Liberia 2022–2023

**DOI:** 10.1017/ash.2025.10077

**Published:** 2025-08-22

**Authors:** Ronan F. Arthur, Ashley Styczynski, Krithika Srinivasan, Amos Tandanpolie, Philip Bemah, Ethan Bell, Jason R. Andrews, Tom Baer, Jorge L. Salinas

**Affiliations:** 1 School of Medicine, Stanford University, Palo Alto, CA, USA; 2 Department of Infectious Diseases, Infection Control, and Employee Health, MD Anderson Cancer Center, Houston, TX, USA; 3 Partnership for Research on Vaccines and Infectious Diseases in Liberia, Liberia; 4 National Public Health Institute of Liberia, Monrovia, Liberia; 5 Stanford Photonics Research Center, Stanford University, Palo Alto, CA, USA

## Abstract

**Objective:**

To evaluate the effectiveness and acceptability of ventilation interventions in naturally ventilated hospitals in Liberia.

**Design:**

Difference-in-differences analysis of pre- and post-air changes per hour of intervention and control spaces.

**Setting:**

Hospitals in Bong and Montserrado Counties, Liberia.

**Participants:**

Seventy patient care spaces were evaluated at baseline. Six spaces underwent physical intervention modifications, while 2 spaces were assessed for indirect effects and 2 others used as controls. Healthcare workers were interviewed to assess ventilation knowledge and acceptability.

**Interventions:**

Ventilation interventions included the installation of window screens, louvered doors and windows, and wind turbines.

**Methods:**

We measured carbon dioxide levels with portable meters and documented persons per room to estimate per-person ventilation rates in both L/s/person for the initial assessment and air changes per hour (ACH) in the intervention. Measurements were taken in patient care spaces in 7 hospitals in Liberia. Healthcare worker acceptability was evaluated via structured interviews.

**Results:**

Two-thirds (46/70) of patient care spaces were below the WHO-recommended ventilation threshold of 60 L/s/person. Six spaces underwent ventilation interventions, including placement of window screens (3), wind turbines (2), and louvered doors and windows (1), with 2 additional spaces being indirectly affected by these interventions and 2 more spaces serving as controls. Ventilation improved by an average of 2 ACH in the spaces with wind turbines and louvered doors and windows. Overall acceptability of the interventions was high.

**Conclusions:**

Implementing interventions to improve ventilation in naturally ventilated healthcare facilities is efficacious, feasible, and acceptable, though longer-term evaluations should assess sustainability.

## Background

Many healthcare facilities in low-resource settings are under-equipped to prevent respiratory disease spread among patients or healthcare workers. Factors such as multiple-bed wards that are common in such settings can increase the risk for respiratory disease spread.^1^ Ventilation is a critical pillar of infection prevention and control (IPC) for preventing respiratory disease transmission. Healthcare-associated transmission of SARS-CoV-2, for example, was found to correspond with facilities having poorer ventilation.^2^ IPC programs typically focus on interventions such as hand hygiene, use of personal protective equipment, and environmental cleaning, while ventilation is less prioritized.^3^

Mechanical ventilation is generally recommended in healthcare settings for its filtration capabilities, consistency, and efficiency.^4^ Yet, many hospitals in low- and middle-income countries (LMICs) cannot afford the installation costs, do not have continuously available electricity to power mechanical ventilation systems, and do not have access to trained personnel to perform the required maintenance.^5^ Optimized natural ventilation has been found to perform at least as well as, if not better than, mechanical ventilation in promoting air exchange and reducing infectious disease transmission risk.^6–8^ Moreover, contaminated mechanical ventilation systems have been implicated in the spread of nosocomial infections, including pathogens not typically regarded as respiratory pathogens.^9–12^ Thus, mechanical ventilation may not be a panacea for solving ventilation in low-resource healthcare settings, particularly those without extreme temperature fluctuations.

Rebreathed air and, as an extension, the risk of indoor airborne disease transmission, can be measured and inferred from indoor carbon dioxide (CO_2_) concentration.^13–14^ These techniques have been used to optimize ventilation in naturally ventilated healthcare facilities in LMICs to better protect against airborne disease transmission.^6–7,15^ Augmentation of ventilation in naturally ventilated spaces can be achieved through building modifications or assistive devices. Strategies for improving ventilation in naturally ventilated healthcare facilities can range from opening existing windows and doors to more complex mixed-mode (hybrid) ventilation strategies that employ mechanical fans; the effectiveness of these various interventions depends on structural constraints, weather patterns, and electricity access.^5,16^

Liberia is considered one of the most vulnerable countries in the world to infectious disease. Frontline healthcare facilities are the first to face novel cases of respiratory and emerging diseases, placing patients and healthcare workers at risk and often leading to disease amplification during outbreaks.^17–18^ Pathogens transmitted in hospital settings through the air pose a particular threat because they can be easily dispersed between infected and uninfected patients via shared airspaces when ventilation is inadequate.^5^

Few low-cost ventilation improvements have been deployed in LMIC healthcare facilities, and little is known about what interventions may be most cost-effective, acceptable, and effective for improving ventilation with limited resources. We aimed to evaluate ventilation in Liberian healthcare facilities, identify and implement contextually appropriate low-cost ventilation interventions, and evaluate the impact of the interventions to improve ventilation and strengthen the resiliency of healthcare facilities.

## Methods

### Study design

The study was conducted across two phases: Phase 1 involved primary data collection with a baseline ventilation assessment to determine appropriate locations for intervention, while Phase 2 consisted of physical ventilation interventions as well as measurements of impact and acceptability among hospital staff.

### Phase 1: Hospital baseline ventilation assessment

Cross-sectional ventilation measurements were collected in patient care areas in hospitals in both urban and rural areas of Montserrado and Bong Counties in Liberia. Hospitals were selected in collaboration with the National Public Health Institute of Liberia and the Ministry of Health of Liberia. Ventilation measurements were performed using portable CO_2_ meters (AmprobeⓇ CO2-100 CO_2_ meter, Amprobe Test Tools, Everett, WA) to measure levels of indoor and outdoor CO_2_, temperature, and humidity. In a single visit, the number of individuals present in a closed space was recorded once, and the existing ventilation infrastructure was documented. CO_2_ levels were collected twice per room in accordance with WHO-recommended protocols.

Absolute ventilation (L/s) was estimated using CO_2_ concentration differentials between indoor and outdoor air, following standard methodologies.^9,13^ Ventilation per person (L/s/p) was then calculated and compared to the WHO-recommended threshold of 60 L/s/p for general patient wards.^5^

### Phase 2: Hospital ventilation infrastructure interventions

Hospitals with inadequate ventilation were selected for intervention in collaboration with national health authorities. Through review of the literature and discussion with ventilation experts, we identified potential ventilation interventions for enhancing natural ventilation in each space selected. We assigned specific interventions to individual spaces based on logistical considerations, including physical infrastructure, geography, and availability of materials, as well as anticipated impact on ventilation. Construction personnel were consulted and contracted to complete the installation of designed physical interventions and for the acquisition or fabrication of required materials. Materials not easily found available in Liberia (ie, whirlybird turbine vents) were procured in the United States.

Existing ventilation features, such as open brick structures for cross-ventilation, had been sealed off in one of the hospitals (Supplementary Figure 2). Neither hospital had negative pressure capacities.

#### Healthcare worker interviews

To assess healthcare worker knowledge about ventilation, identify ventilation challenges, and solicit input for ventilation improvements, we conducted open-ended interviews with a subset of healthcare workers in the selected hospitals using convenience sampling. Interviewers asked about knowledge of ventilation, importance of ventilation, and questions about doors, windows, and air-conditioning, including preferences and suggestions. Interviews were recorded and transcribed, and key themes were identified using constant comparison analysis and grounded theory.^19^

#### Assessment of intervention impact

Longitudinal CO_2_ measuring devices (Aranet4, SAF North America LLC, Aurora, CO) were placed in each of the intervention and control spaces. Baseline CO_2_ values were measured in 5-minute intervals over 1 week. To assess the average number of people in the space, research associates visited each space at different times of day. The volume (m^3^) of the space was calculated by laser measurements. Following the implementation of the interventions, CO_2_ measurements were again collected over a week in both the intervention and control spaces. We calculated absolute ventilation as above, which we converted to air changes per hour (ACH) based on room volume (m^3^).

To assess the impact of the physical interventions on ACH in treatment versus control spaces, we employed a difference-in-differences framework, whereby the difference between pre- and post-intervention phases for direct (louvered doors and whirlybirds), indirect (whirlybird-adjacent), screen, and control arms was assessed. The analysis utilized a Gaussian linear mixed-effects regression model to account for data hierarchy. Fixed effects included time (pre- vs post-intervention), intervention group (direct, indirect, screen, and control), and their interaction. Random effects accounted for variation across locations. Residuals from the model were assessed for normality. All statistical analyses were conducted in R, using the lme4 package for mixed-effects modeling.

#### Acceptability assessment

We conducted acceptability assessments of the interventions through surveys and interviews with healthcare workers in the intervention spaces. Healthcare workers were selected through convenience sampling. The questionnaire was an adaptation of a theoretical framework of accessibility for healthcare interventions.^20^

### Institutional review

The Stanford University Institutional Review Board (protocol #67822) and the Atlantic Center for Research and Evaluation Institutional Review Board in Liberia (Assurance #FWA00032198) approved this study.

## Results

### Baseline ventilation assessment

From August 2022 to February 2023, we sampled all patient care areas in 7 hospitals. Measurements collected from one of the hospitals registered indoor CO_2_ values lower than 400 ppm. As this was presumed to be due to a calibration error, these data were dropped from further analysis. The remaining data included 70 patient care areas across 6 hospitals. Of the 70 rooms, 46 (66%) of the spaces were below the ventilation threshold of 60 L/s/person for general patient care activities (Supplementary Figure 1). Notably, there were several instances where existing ventilation infrastructure had been blocked in the setting of air-conditioning unit introductions (Supplementary Figure 2).

### Healthcare worker interviews

Interviews were conducted with 21 healthcare workers from 2 hospitals selected for ventilation interventions, including 13 nurses, 6 nursing supervisors, an IPC focal person, and a midwife. Responses highlighted healthcare worker knowledge and awareness about ventilation as well as existing ventilation challenges within healthcare facilities (Table 1). Some healthcare workers recognized the importance of ventilation, citing it as a means of preventing respiratory disease spread. However, other healthcare workers equated climate control with ventilation, citing air-conditioning as a preferred ventilation modality. Several barriers to opening existing windows and doors were noted, including privacy concerns, concerns over mosquitoes and vermin, air pollution, and countering the cooling effects of air-conditioning. Recommendations for improving ventilation included the addition of window screens and expansion of windows.

**Table 1.** Key themes around ventilation knowledge and challenges among Liberian healthcare workers (*N* = 21).

Theme	Main findings	Key quotes
Knowledge about ventilation	Ventilation protects against infectious risks	“Ventilation…helps to reduce the risk of infection of the patient especially within the hospital setting like where I’m working now.”
		“When the windows and doors are open, the one that can make you feel safer is ventilation because when they come in with a respiratory disease like TB you catering to that patient, the place need to be well ventilated and to open the windows so that good air can come in and bad air goes out.”
Lack of awareness of ventilation	Equating temperature control with ventilation	“I’m telling you now like I said previously—to improve ventilation, to you people now I will say, that we need AC.”
		“On my ward I have air cool, so it is not necessary for the windows and doors to be open.”
		“Actually I will love to see clean environment and all wards be properly ventilated by air conditions, standby fan, and electricity.”
Barriers to opening windows or doors	Privacy concerns	“Okay yes, when the windows are open you don’t have privacy for your patient, because everything that will be said in your assessment area, passerby will hear them, so sometimes we like to see our patients close door and say what between us.”
	Competition with air-conditioning	“Yes, right now we are not opening doors and windows because of the AC and fan.”
	Concerns over insects and other vermin	“Because, most often especially overnight, mosquitoes and other things you can’t open the window when you have babies.”
	Concerns over air pollution	“Air that is coming in windows and doors are all open air that is coming in is polluted, I will not feel safe to work in that kind of place.”
Suggestions to improve ventilation	Expansion of windows	“Expansion of windows by creating another window elsewhere.”
	Addition of window screens	“Like if the window have a screen all around it will prevent insects and other things from entering but just open window, harmful things will enter and it will not be safe.”

### Review of ventilation strategies

Six patient care spaces in 2 hospitals were selected for ventilation interventions with 2 additional spaces to serve as controls. Because of the physical layout of the rooms, 2 rooms were incompletely sealed from adjacent care areas. These adjacent rooms were assigned as “indirect” intervention spaces. Six potential ventilation interventions were considered for possible implementation (Table 2). Given structural considerations and availability of materials, 1 space was selected for placement of louvered windows and doors with screens, 2 spaces were selected for placement of wind turbines, and 3 spaces were selected for placement of window screens. Two adjacent rooms were indirectly served by the wind turbines (Supplementary Figure 3). Although window screens do not intrinsically improve ventilation, it was thought that cross-ventilation could be enhanced by addressing key barriers to keeping windows open (eg, mosquitoes) through placement of window screens. Cost was an additional consideration in selection of interventions as a key aim of the intervention was to identify solutions that would be feasible in LMICs. Healthcare workers in each of the spaces were sensitized to the interventions and counseled on the importance of ventilation for reducing the risk of transmission of airborne infectious diseases to both patients and healthcare workers.

**Table 2.** Ventilation modification strategies to enhance natural ventilation.

Strategy	Anticipated cost	Requires structural modifications	Requires behavioral inputs	Requires electricity	Other considerations
Cross ventilation via open windows/doors	$–$$	+/–	+	–	• May be difficult to employ continuously because of temperature, climate, or security concerns. • May not keep out insects or vermin unless combined with screens. • Most effective when windows or doors are on opposite walls and on windward/leeward sides of the building.
Louvered windows/doors	$$	+	+/–	–	• Can usually be left open during rain. • Can be engineered to prevent full closure. • May not keep out insects or vermin unless combined with screens. • Provides less ventilation than a fully open window/door.
Honeycomb bricks/vents	$$	+	–	–	• Can also produce an added cooling effect when placed near the top of the space via stack effect. • May not keep out insects or vermin unless combined with screens.
Wind turbines/wind tower	$$$	+	–	–	• Requires constant breeze to be effective.
Chimney/stack tower	$$$$	+	–	–	• Dependent on temperature differential, can be augmented with solar atria. • Many possible configurations.
Exhaust fans	$$$	–	–	+	• Requires ‘sealing’ of the space to obtain significant air draw. • Can be used to create negative pressure and ensure outward flow of air.

Wind turbines were placed in single-story buildings located in relative proximity to the ocean to capitalize on the continuous breeze. Galvanized wind turbines were selected to prevent accelerated rusting in a high salinity context. The wind turbines were not available locally and had to be imported. Four 14-inch diameter turbines were distributed over the roof of the 2 adjacent spaces, which was anticipated to improve the ventilation by up to 4 ACH in each of the spaces. During placement, it was noted that there was a 2–3-foot gap between the roofline and the ceiling. Metal sheeting was used to create a conduit connecting the wind turbine to the ceiling (Supplementary Figure 4).

Louvered windows and doors and screens were manufactured locally. The louvered windows and doors were designed to prevent them from being fully shut to ensure at least some degree of continuous cross-ventilation (Supplementary Figure 5). Screens were overlaid on these windows and doors, which were placed on opposite walls in a space that was on the first floor of a multi-storied building.

### Assessment of intervention impact

**Figure 1.**
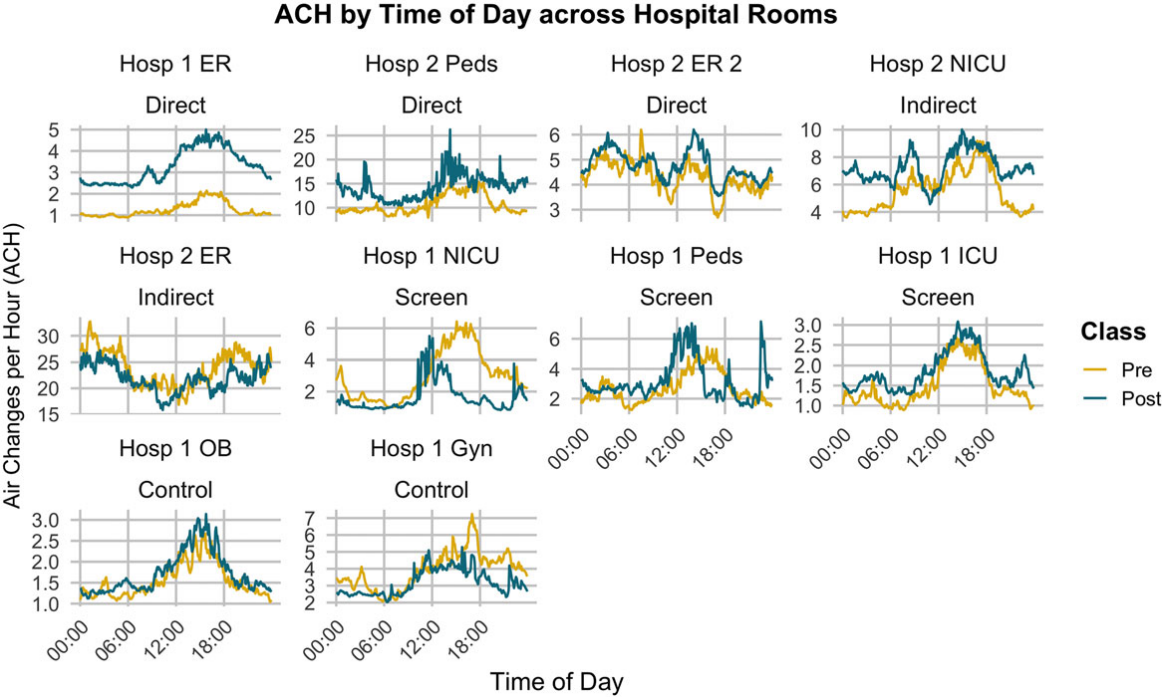
Air changes per hour (ACH) by time of day across all hospital rooms pre- (yellow) and post- (green) intervention installation. Each hospital space was designated an intervention type: direct (immediate interiors to infrastructure changes), indirect (proximate interiors), screen (immediate interiors to screen installations), and control (no changes).

At baseline, each of the selected spaces was noted to have ventilation rates below the WHO threshold. Substantial variability was noted in CO_2_ values by time of day, and ACH values in both pre- and post-data align with this observation (Figure 1). Each intervention type (direct, indirect, screen, and control) had a distinct impact on ACH measurements compared with preintervention measurements (Table 3). Direct interventions (ie, the immediate interiors where louvered windows, doors, and/or whirlybird installations were made) had the highest levels of improvement after installation of any intervention type (average ACH increases from .45 to 3.60). Hospital 2 ER, one of the 2 indirect interventions (ie, the spaces proximate to infrastructure improvement), demonstrated little effect from the intervention (ACH decrease of 2.0), though ventilation was more than adequate both before and after (overall average ACH of 22.91). The other space demonstrated substantial improvement only at night (overall ACH increase of 1.69). The greatest improvement was seen in the space where louvered windows and doors were added (3.29 average ACH at post vs 1.26 ACH prior to intervention). Some of the screen interventions also performed better at night compared with baseline, while one performed more poorly after the intervention. The controls saw little change to ACH measurements (average ACH changes of .18 and −.63).

**Table 3.** Comparison of pre- and post-intervention air changes per hour (ACH) in each intervention and control ward space.

Location	Intervention type	Intervention	PreACH IQR	PostACH IQR	Δ ACH
Hosp 1 ER	Direct	Louvered doors/windows	(.65–1.49)	(1.72–4.45)	2.03
Hosp 2 Peds	Direct	Whirlybird	(7.9–12.43)	(9.23–16.64)	3.6
Hosp 2 ER 2	Direct	Whirlybird	(3.63–4.96)	(3.49–5.53)	.45
Hosp 2 NICU	Indirect	Whirlybird-adjacent	(3.65–6.74)	(5.45–8.68)	1.69
Hosp 2 ER	Indirect	Whirlybird-adjacent	(18.4–27.6)	(17.56–25.28)	−2.0
Hosp 1 NICU	Screen	Screen	(1.12–4.32)	(.98–1.75)	−1.35
Hosp 1 Peds	Screen	Screen	(1.34–3.41)	(2.1–3.51)	.49
Hosp 1 ICU	Screen	Screen	(.75–1.65)	(.97–2.48)	.33
Hosp 1 OB	Control	Control	(1.06–1.84)	(1.15–2.01)	.18
Hosp 1 Gyn	Control	Control	(2.69–4.8)	(2.19–3.86)	.63

Results from the linear mixed-effects model (Table 4) indicate that the improvements in ACH following the direct infrastructure interventions were substantial and significant compared to control spaces, adjusting for pre-intervention ACH levels (*β* = 2.30, *P* < .001). By contrast, changes in ACH in the indirect and screen interventions were not significant from the pre- to post-intervention phases.

**Table 4.** Results from the Gaussian linear mixed-effects model.

Term	Estimate	95% CI	*P*-value
Baseline (intercept)	2.73	(−4.05, 9.51)	.50
Post-intervention (class)	−.27	(−.45, −.09)	<.01
Direct intervention	2.68	(−6.07, 11.44)	.51
Indirect intervention	12.0	(2.41, 21.59)	<.05
Screen intervention	.32	(−9.08, 8.44)	.94
Scaled time (truncated)	.22	(.18, .26)	<.001
Interaction: post × direct	2.3	(2.07, 2.53)	<.001
Interaction: post × indirect	.12	(−.14, .37)	.34
Interaction: post × screen	.08	(−.15, .31)	.49

## Discussion

Ventilation represents a key vulnerability for healthcare IPC in LMICs. Most healthcare delivery spaces evaluated in Phase 1 had lower than recommended ventilation rates at baseline. The impact of infrastructure interventions on ventilation rates in Phase 2 was variable with some spaces demonstrating substantive improvements, while other spaces showed no significant differences compared to baseline.

Ventilation inadequacies and challenges in Liberian hospitals are reflected in other low-resource settings.^15,21^ Healthcare workers demonstrated variable understanding of the importance of ventilation for reducing infectious disease transmission. This may at least partially explain the large number of spaces with inadequate ventilation across 6 hospitals. The finding that some clinical staff viewed ventilation as relatively unimportant is reflected in the literature.^22^

A major competing priority in many spaces was climate control (i.e. air-conditioning) as evidenced by the sealed-off remnants of prior ventilation infrastructure observed during baseline assessments. The importance placed on air-conditioning was also highlighted during interviews with staff whose main focus was climate control, which was often equated with ventilation or considered to be at odds with ventilation (e.g. keeping windows shut when the air-conditioning is running). The air-conditioning units typically placed in these spaces recirculate cooled air rather than drawing in external air, and, therefore, do not contribute to ventilation. The increasing use of air-conditioning to decrease temperature but without an impact on air exchanges is particularly problematic for infectious disease transmission risk.^23^

Yet, ventilation and climate control do not have to be at odds. In fact, thresholds for ACH in healthcare settings were largely driven by their impact on patient comfort.^24^ Many ventilation interventions provide both improved ventilation as well as climate control through continuous air movement and/or removal of hot air through stack effect. Interventions relying on stack effects may have greater impact on climate control, though these interventions were not undertaken in this study because they would have required more expensive structural modifications, and it was unclear if temperature differentials were sufficient to achieve the desired outcome. If further climate control is required, augmented ventilation systems can be employed.^25–27^ Furthermore, maintaining open windows or doors while running air-conditioning units is thought to impede the impact of climate control and substantially raise energy costs. However, these may not be as oppositional as anticipated, as the estimated additional energy costs associated with operating air-conditioning while maintaining partially open windows are in the order of $6/month (assuming energy costs of $.15 USD/KwH).^28^

In this study, several of the spaces were observed to have varying ventilation when assessed by time of day. In general, ventilation rates were lower at night, presumably driven by closing windows and doors. This likely explains the limited impact of screens. Ventilation rates were highest during the afternoon, however, presumably driven by opening windows and doors for relief from higher temperatures. However, the time of open windows was not measured in this study, so the lack of impact may be related to a lack of change in practice rather than a failure of the intervention. Window screens also did not address 2 other stated concerns—privacy and air pollution—which may have discouraged any change in window opening patterns.

The wind turbines were anticipated to have the most appreciable impact on ventilation, though the observed changes were more modest. One key reason may be because of the large gap between the roofline and the ceiling, which necessitated crafting of air conduits. Flow decreases as the length of a cylinder increases, so the effect may have been dampened by the added length of the conduit. Additionally, the team had planned to install twice as many turbines over the designated spaces, but there were changes in the hospital administration that prevented approval for the installation of all the turbines. Turbines may still have strong potential for low-cost ventilation solutions in hospital settings as they can both circulate air and provide a cooling effect by withdrawing hot air from the ceiling space.

Limitations of this study include the small number of spaces and interventions that were included in the assessment, which limits the generalizability of the specific interventions. Additionally, we did not assess long-term sustainability. If staff perceived the interventions as reducing the impact of climate control, the interventions could be reversed, such as with the sealed honeycomb bricks, which could undermine the ventilation improvements we documented in that space. Moreover, while CO_2_ measurements were collected every 5 minutes by an automatic sensor, the measurement of people in the room was done by research personnel more infrequently and never in the middle of the night—thus, the number of people in the room may have been overestimated as nighttime traffic through the hospitals was likely less than during the day. Furthermore, we did not assess process measures, such as time of open windows or other behavior changes, which would more accurately characterize impact of interventions such as window screens.

Building resilient healthcare systems to prepare for future epidemics requires practical, cost-effective solutions that align with local cultural and resource constraints. Our findings demonstrate the feasibility of implementing natural ventilation interventions in Liberian healthcare facilities. These modifications were both efficacious and acceptable, though longer-term evaluations are necessary to assess sustainability. Future research should explore cost-effectiveness, feasibility in other low-income settings, and integration into broader infection prevention strategies to ensure that these interventions are both impactful and scalable.

## Supporting information

10.1017/ash.2025.10077.sm001 Arthur et al. supplementary material Arthur et al. supplementary material

## Supplementary material

The supplementary material for this article can be found at https://doi.org/10.1017/ash.2025.10077.
