# Supplementary material for: Effectiveness and acceptability of ventilation modifications in healthcare facilities, Liberia 2022–2023
Source: Antimicrob Steward Healthc Epidemiol. 2025 Aug 22;5(1):e190. doi: 10.1017/ash.2025.10077 (PMC12394025; doi:10.1017/ash.2025.10077)
Supplement: Arthur et al. supplementary material [file S2732494X25100776sup001.docx]

# **Supplementary Materials**

#
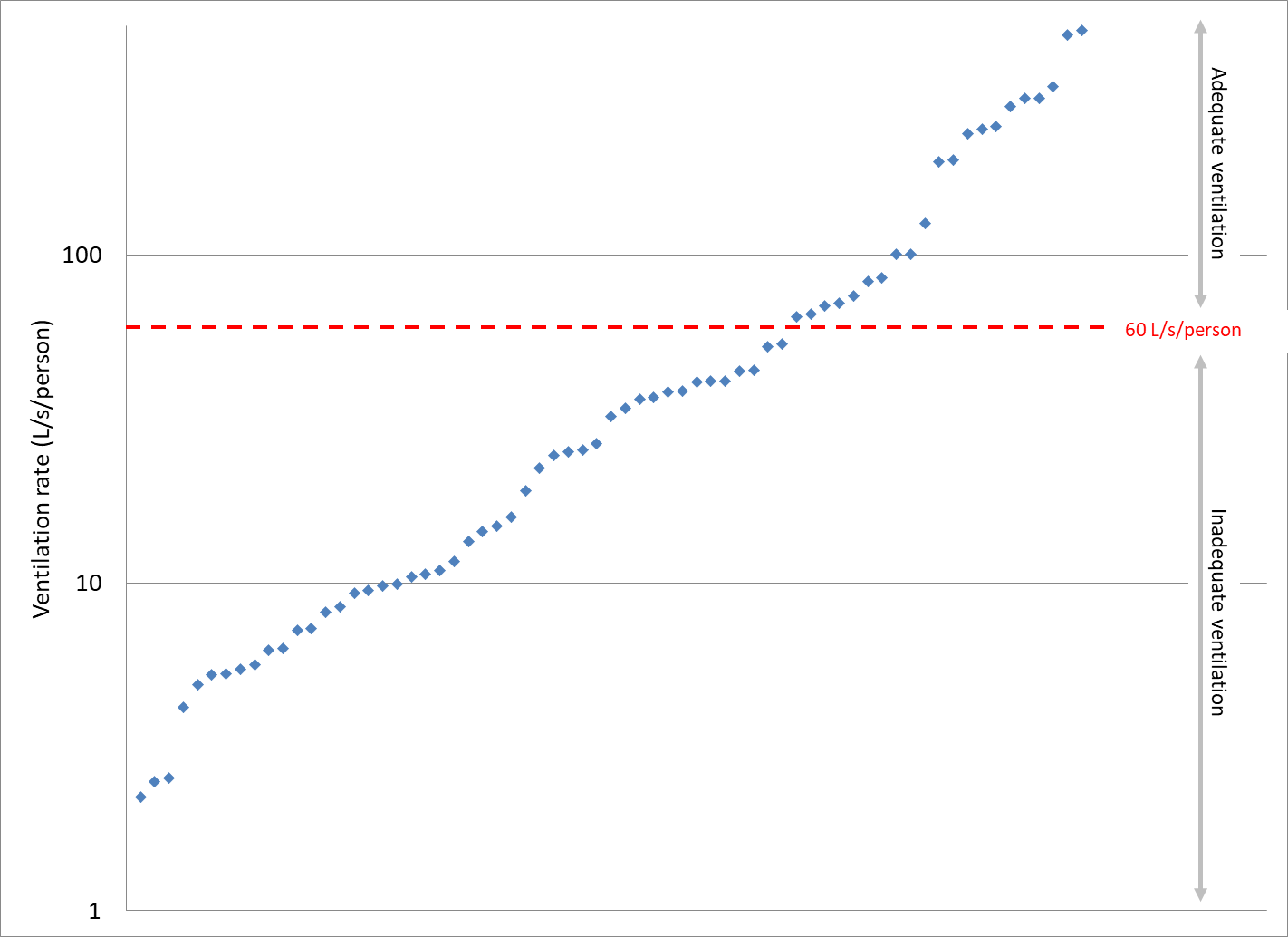


Supplemental Figure 1. Baseline ventilation rate of patient care spaces across 6 Liberian hospitals, 2022-2023 (N=70)


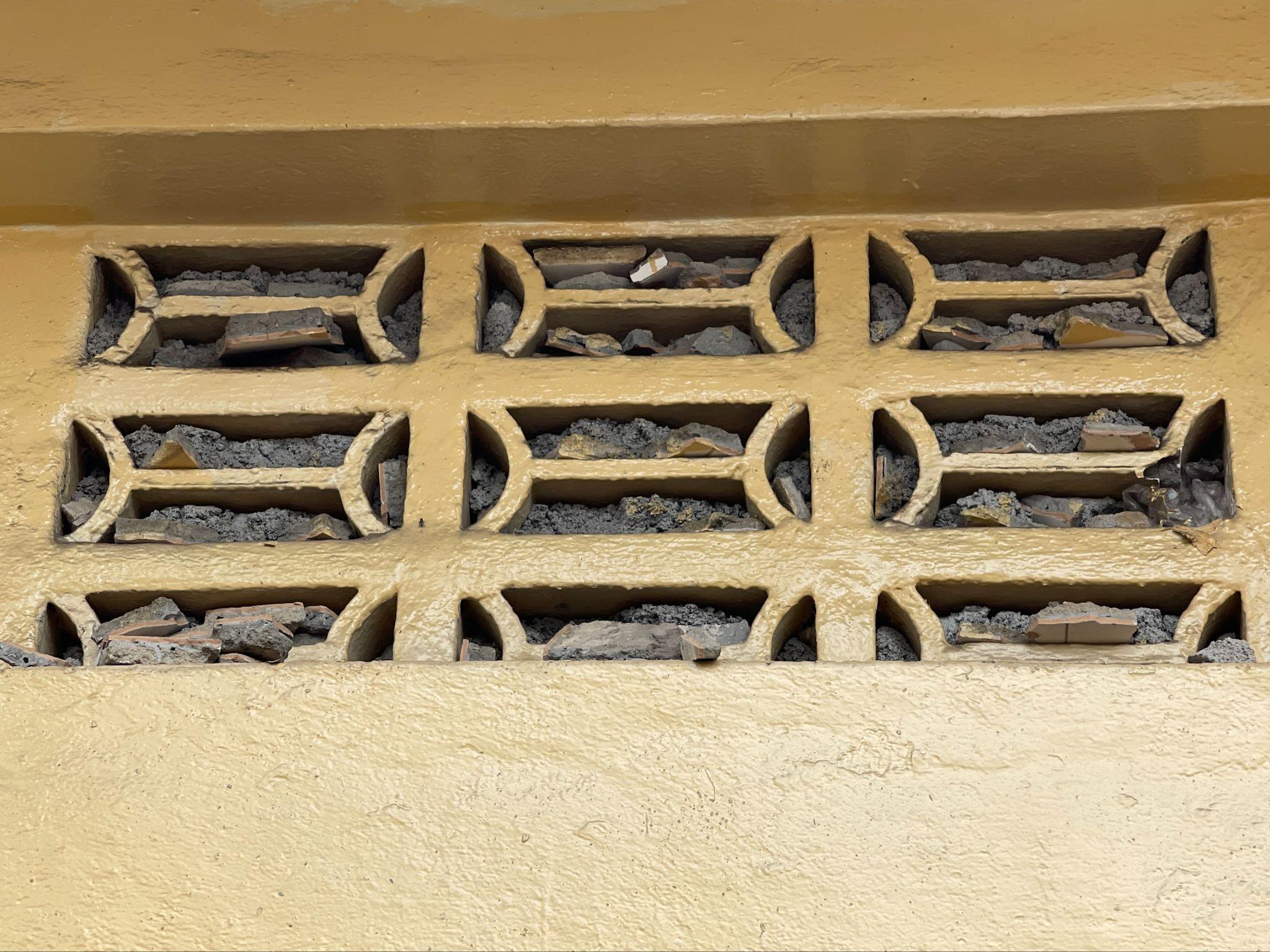


Supplementary Figure 2. Photo of previous ventilation infrastructure having been sealed.


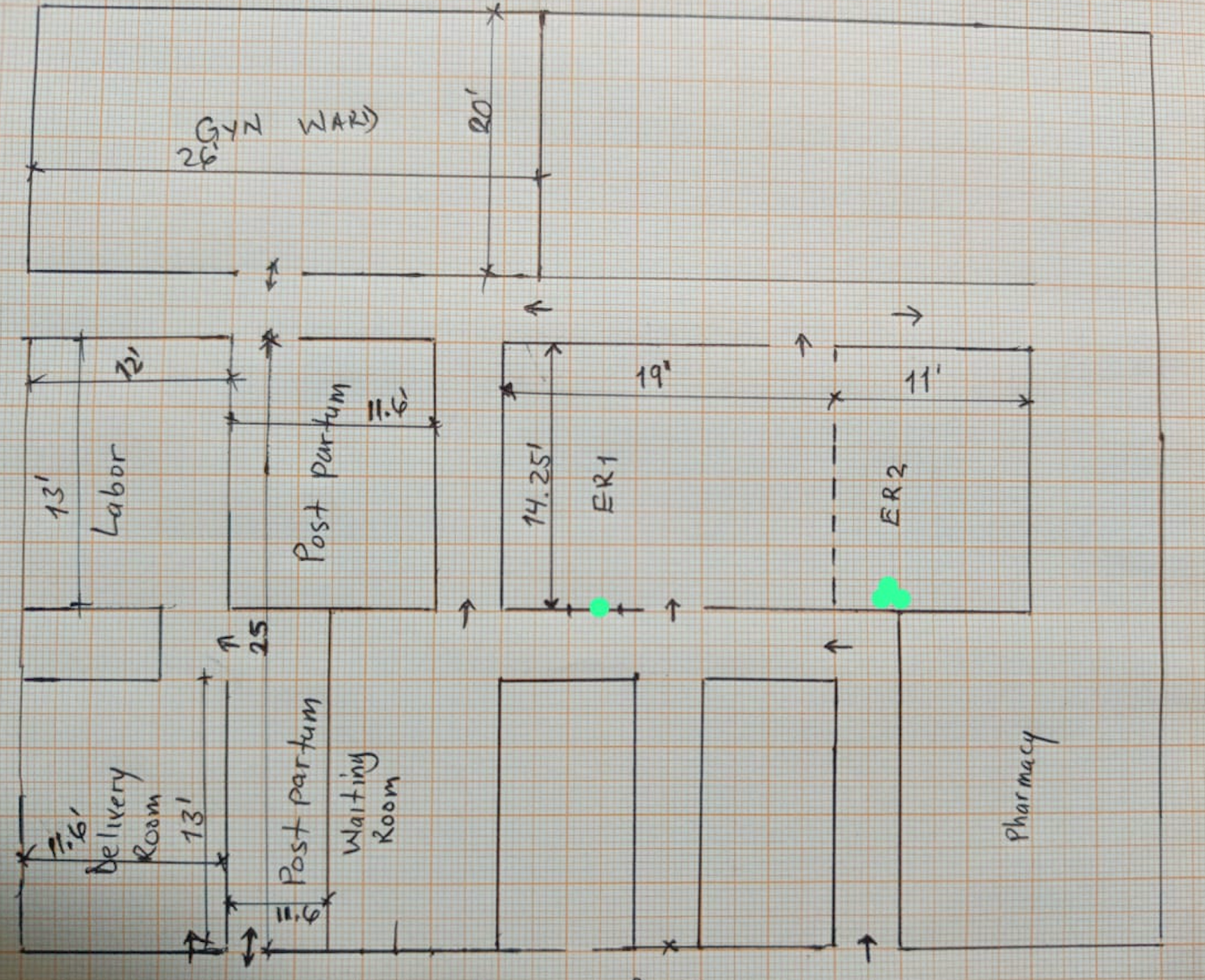


Supplementary Figure 3: Floor plan schematic for Hospital 1. Green dots indicate placement of louvered doors and windows.


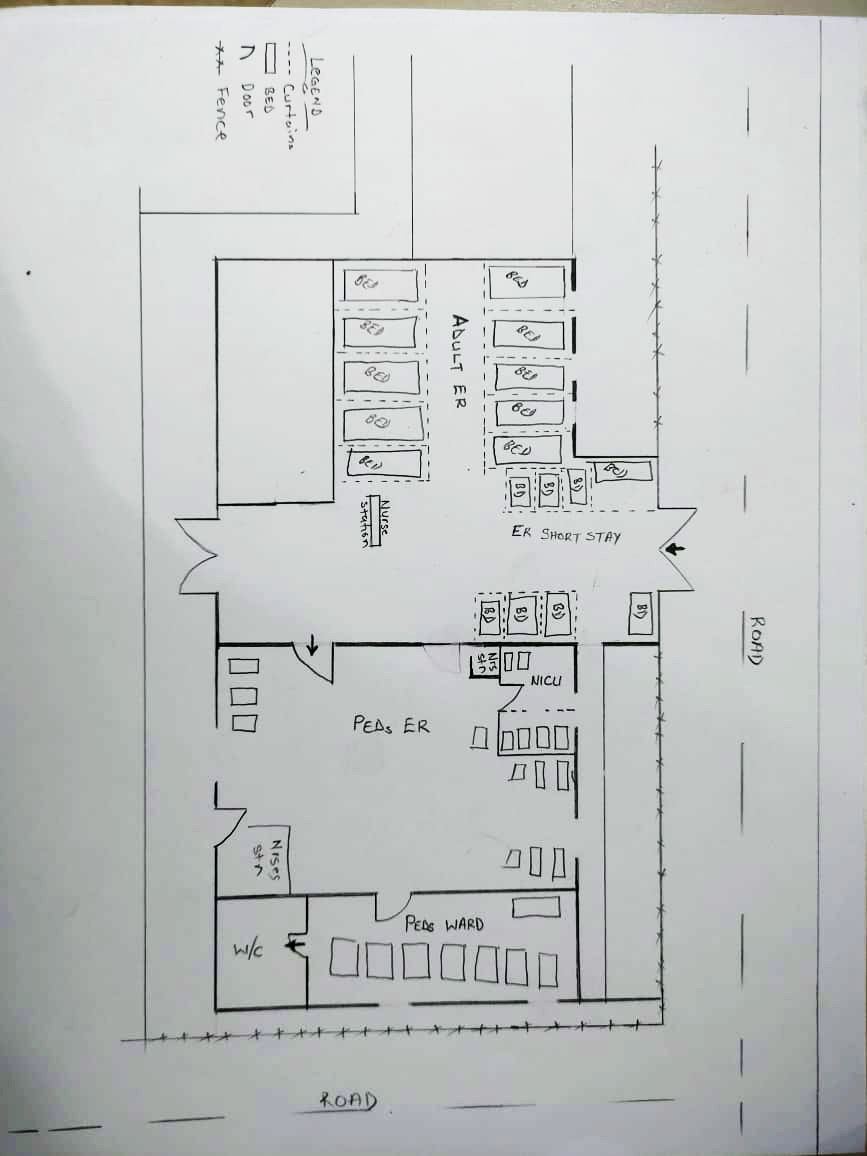


**1**

**2**

**4**

**3**

Supplementary Figure 4: Schematic of whirlybird placement relative to patient care spaces in Hospital 2. Red circles denote locations of wind turbines; blue boxes with numbers indicate patient care areas. Areas 1 and 3 were considered direct intervention rooms; areas 2 and 4 were considered indirect intervention rooms.


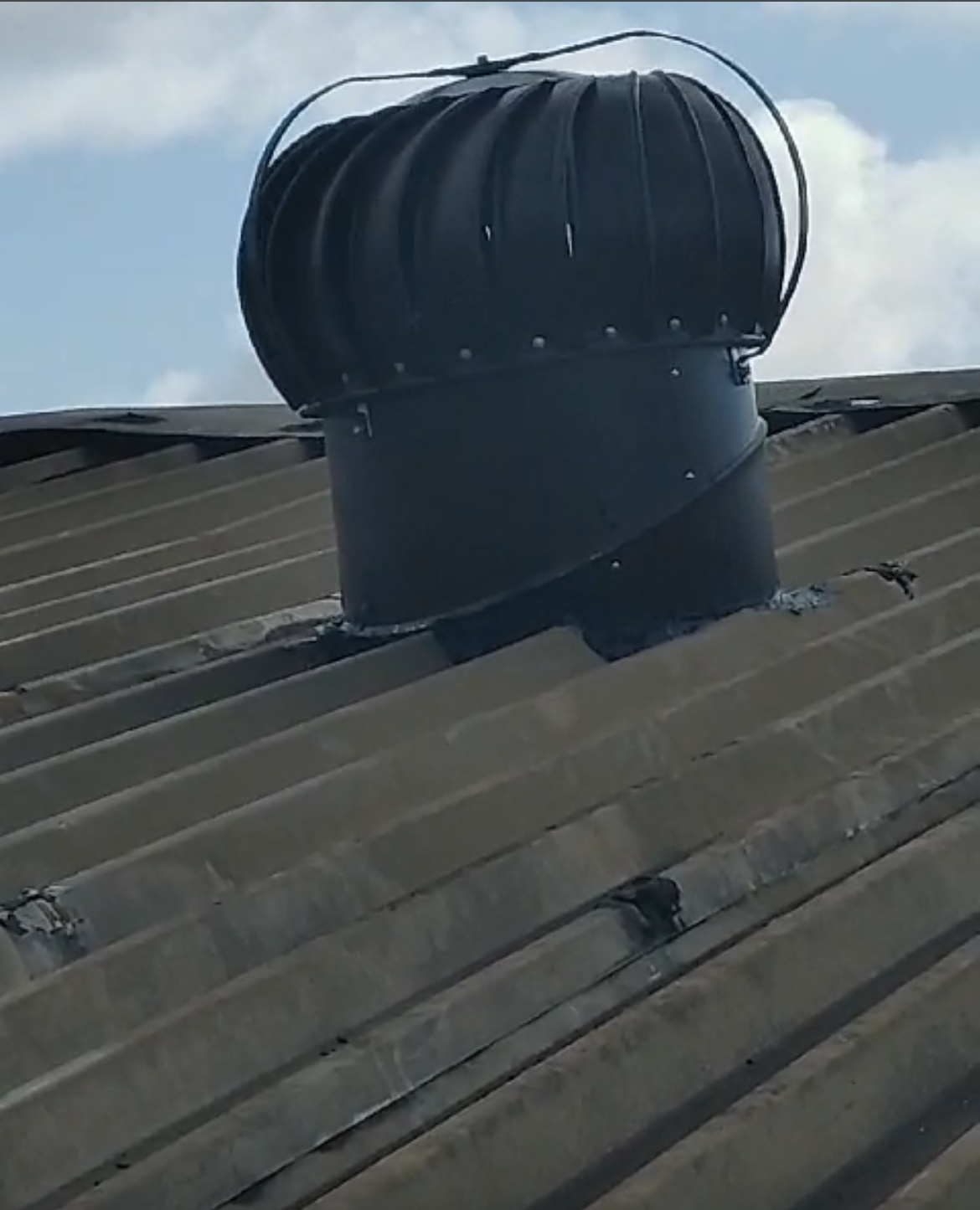

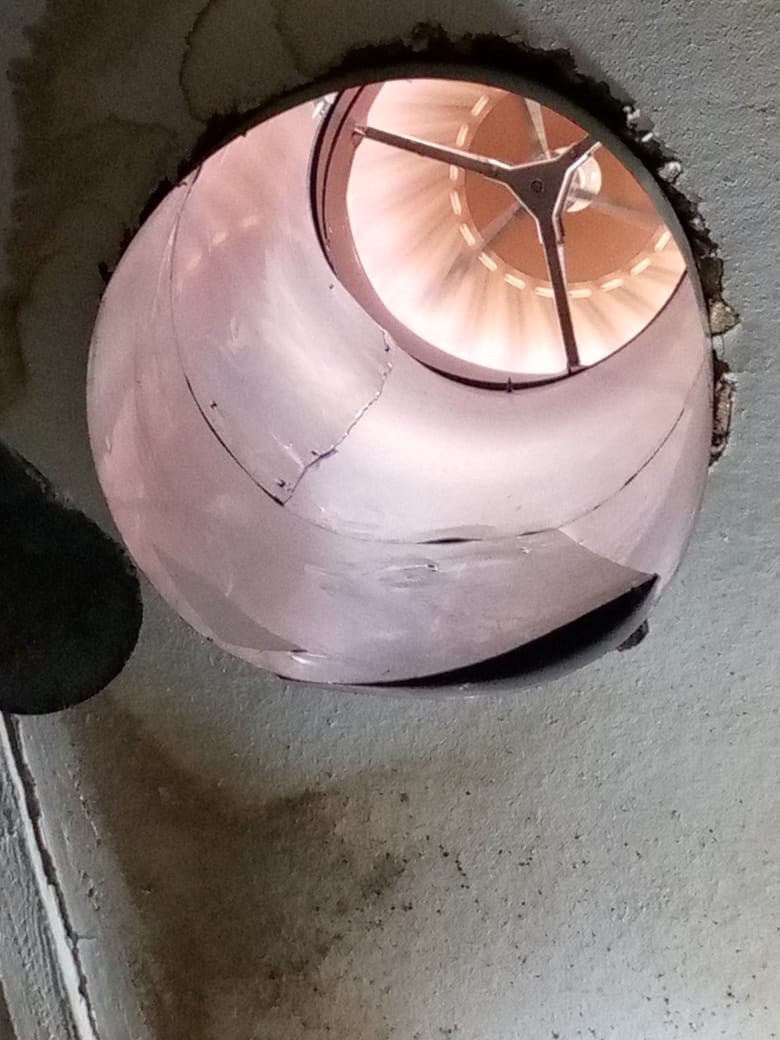


Supplementary Figure 5. Photos of whirlybird installation.


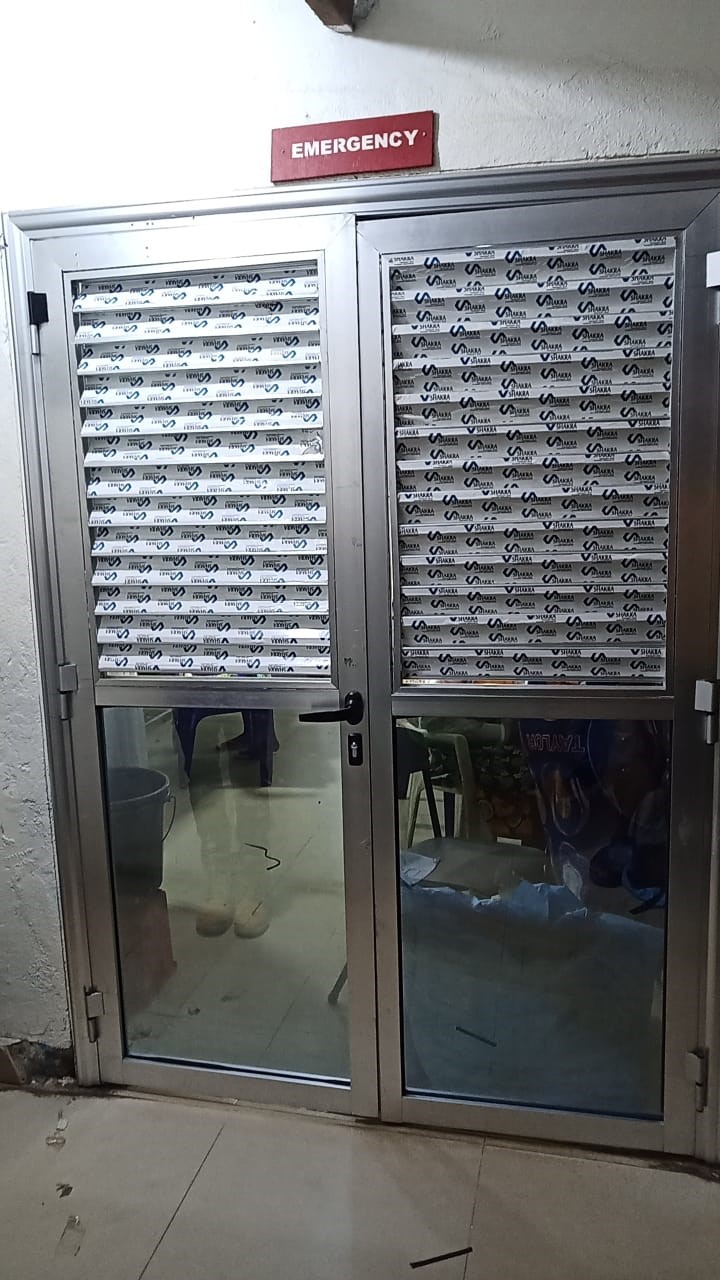

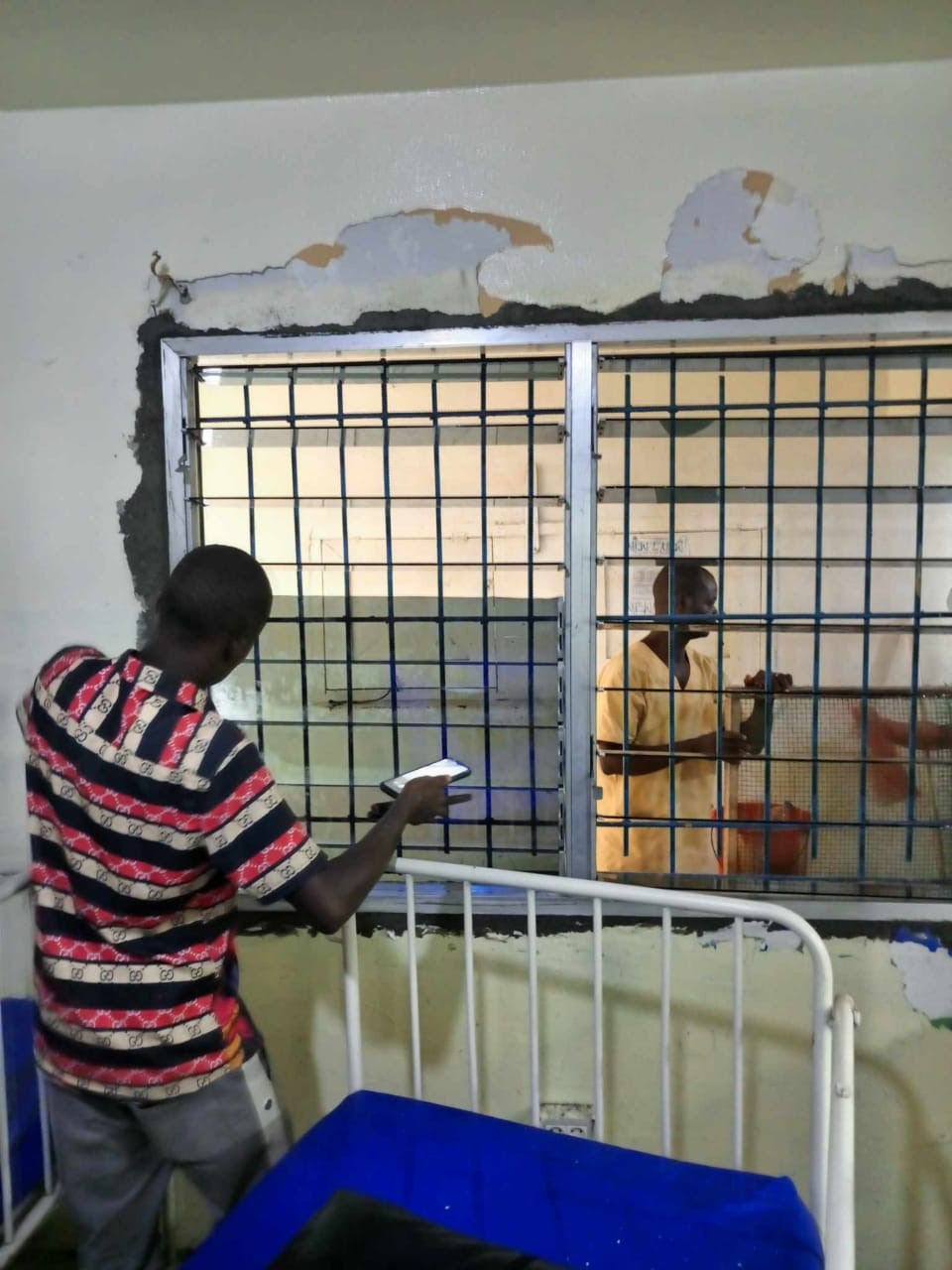


Supplementary Figure 6. Photos of louvred windows and doors installation. Prior to the installation, these doors and windows were pure glass.


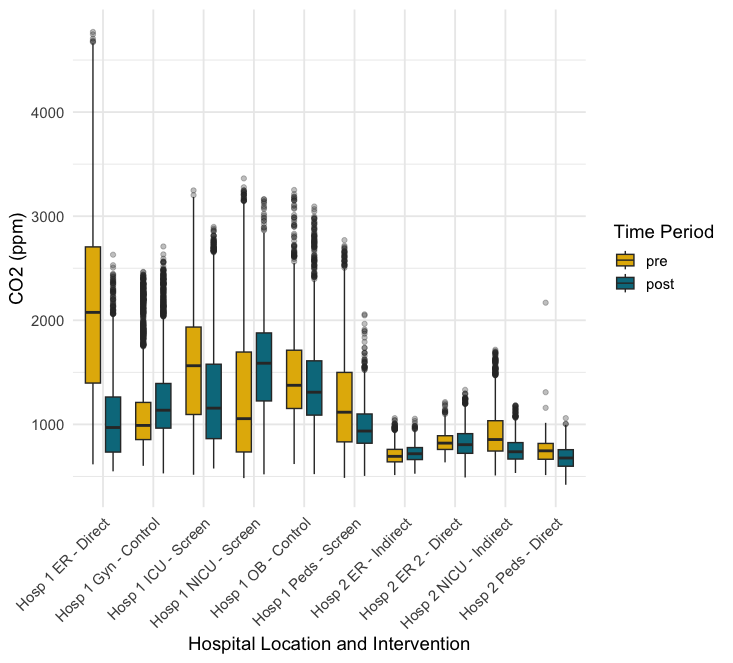


Supplementary Figure 7. Box-and-whisker plots of pre vs. post-CO2 across hospital rooms.


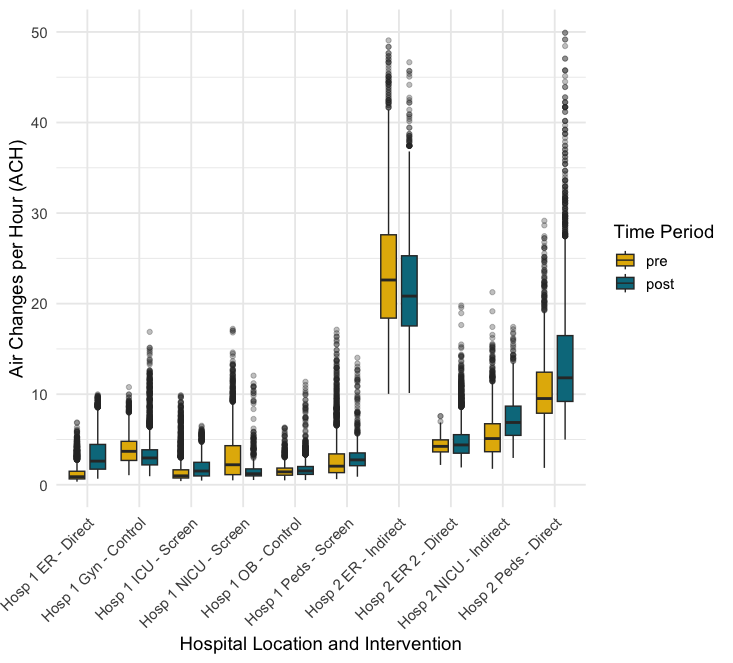


Supplementary Figure 8. Box-and-whisker plots of pre vs. post-ACH across hospital rooms.

Supplemental Table 1. Acceptability survey responses following ventilation interventions in Liberian healthcare facilities (N=15)

| Question | N (%) |
| --- | --- |
| It is clear to me how good ventilation protects the health and well-being of the staff and patients at the hospital. |  |
| Strongly agree | 10 (66.7) |
| Agree | 4 (26.7) |
| No opinion | 1 (6.7) |
| Disagree | 0 |
| Strongly disagree | 0 |
| It is worth making an extra effort to ensure adequate ventilation in the hospital ward where I work. |  |
| Strongly agree | 10 (66.7) |
| Agree | 5 (33.3) |
| No opinion | 0 |
| Disagree | 0 |
| Strongly disagree | 0 |
| Did you like or dislike the ventilation intervention? |  |
| Strongly like | 4 (26.7) |
| Like | 9 (60) |
| No opinion | 2 (13.3) |
| Dislike | 0 |
| Strongly dislike | 0 |
| How fair is the intervention for patients and healthcare workers? |  |
| Very fair | 8 (53.3) |
| Fair | 6 (40) |
| No opinion | 1 (6.7) |
| Unfair | 0 |
| Very unfair | 0 |
| The intervention has improved ventilation. |  |
| Strongly agree | 8 (53.3) |
| Agree | 6 (40) |
| No opinion | 1 (6.7) |
| Disagree | 0 |
| Strongly disagree | 0 |
| It is clear to me how the intervention will help improve ventilation. |  |
| Strongly agree | 6 (40) |
| Agree | 9 (60) |
| No opinion | 0 |
| Disagree | 0 |
| Strongly disagree | 0 |
| How confident do you feel about the intervention? |  |
| Very confident | 6 (40) |
| Confident | 9 (60) |
| No opinion | 0 |
| Unconfident | 0 |
| Very unconfident | 0 |
| The intervention interfered with my other priorities. |  |
| Strongly agree | 0 |
| Agree | 1 (6.7) |
| No opinion | 0 |
| Disagree | 8 (53.3) |
| Strongly disagree | 6 (40) |
| How acceptable was the intervention to you? |  |
| Completely acceptable | 8 (53.3) |
| Acceptable | 7 (46.6) |
| No opinion | 0 |
| Unacceptable | 0 |
| Completely unacceptable | 0 |
